# Fractionation of Wood Biomass With Thiolactic Acid and Choline Chloride‐Based Solvent Into White Lignin for Sustainable Cooling Applications

**DOI:** 10.1002/cssc.202502104

**Published:** 2025-11-28

**Authors:** Juho Antti Sirviö, Mingna Liao, Donya Arjmandi, Jasmiina Haverinen, Ruijie Wu, Magnus P. Jonsson, Chunlin Xu, Ari Ämmälä, Jarkko P. Räty

**Affiliations:** ^1^ Fibre and Particle Engineering Research Unit University of Oulu Oulu Finland; ^2^ Laboratory of Organic Electronics Department of Science and Technology Linköping University Norrköping Sweden; ^3^ Wallenberg Wood Science Center Linköping University Norrköping Sweden; ^4^ Laboratory of Natural Materials Technology Åbo Akademi University Turku Finland; ^5^ Kajaani University Consortium Measurement Technology Unit University of Oulu Kajaani Finland

**Keywords:** biomass, color, green chemistry, lignin, passive radiative cooling

## Abstract

Lignin, a naturally abundant biopolymer, possesses intrinsic ultraviolet (UV) shielding capabilities, making it a promising candidate for sustainable functional materials. However, conventional lignin isolation methods often lead to dark‐colored products due to structural condensation and chromophore formation, limiting its applicability in optical and esthetic applications. In this study, we introduce a novel fractionation strategy utilizing a system composed of thiolactic acid and choline chloride to selectively extract lignin from wood biomass. This environmentally benign process yields a light‐colored lignin with exceptional whiteness (>90%) and high recovery efficiency (70%). The preservation of lignin's bright appearance is attributed to its submicron‐scale morphology and chemical stabilization via thiolactic acid modification, which suppresses chromophore formation. Remarkably, the resulting white lignin demonstrates high visible light reflectance and significantly reduced solar heat gain compared to conventional kraft lignin. Furthermore, its strong UV absorption and high emissivity in the atmospheric transparency window position it as a compelling bio‐derived material for passive radiative cooling applications. This work highlights a sustainable pathway for valorizing lignin into high‐performance, multifunctional materials aligned with green chemistry principles.

## Graphical abstract

A novel and highly efficient wood biomass fractionation method was developed to produce white lignin, exhibiting the brightest color reported to date. The process yielded white lignin with high yield, excellent visible light reflectance, and elevated infrared emissivity, highlighting its potential as an active material for passive radiative cooling applications.

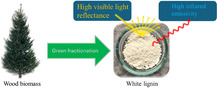

## 1 Introduction

Lignin is the most abundant aromatic polymer on Earth, accounting for ≈30% of terrestrial nonfossil organic carbon [1, 2, 3]. Alongside cellulose and hemicellulose, it constitutes the primary structural component of lignocellulosic biomass, imparting rigidity to plant cell walls and providing protection against environmental impact such as microbial decay [4]. Due to its hydrophobic nature, lignin also plays a role in regulating water transport within plant tissues. Given its abundance and renewability, lignin has been extensively studied as a sustainable alternative to fossil‐derived materials and chemicals [5, 6].

Although native lignin is typically light in color, industrially processed forms—referred to as technical lignin, such as kraft, alkaline, and organosolv lignin—are characteristically dark red to brown. This coloration arises from structural modifications during harsh extraction processes, which involve elevated temperatures, high pressures, and extreme pH conditions [7, 8]. These conditions promote condensation reactions and the formation of conjugated carbonyl structures, increasing chromophore density and light absorption [9]. The incorporation of heteroatoms can further intensify coloration. Consequently, the dark color of residual lignin in cellulose pulp necessitates additional bleaching steps, which are chemically intensive and environmentally burdensome.

To address these challenges, several strategies have been explored to isolate light‐colored lignin to be utilized in applications such as sunscreens [10, 11]. For example, Lewis acid‐based deep eutectic solvents (DESs), as a combination of AlCl_3_ with 1,4‐butanediol and choline chloride, have been used to extract lignin from softwood, yielding materials with high lightness (L^*^ values of 78) [12]. A similar high lightness (L^*^ value of 74) was obtained by synergizing the mitigated spatial confinement and chemical stabilization of lignin using 1,4‐butanediol together with citric acid [13]. Furthermore, lignin with an ISO brightness of 30% was isolated from bamboo using a polyol‐based DES [14]. Other approaches are brightening of technical lignin by solvent‐controlled encapsulation with isocyanates [15, 16] and decolorization via acetylation and UV irradiation [17]. Additional methods involve methanol–water solvent systems [18], ultrafiltration with ultrasonic cavitation [19], and morphological control to produce nanolignin with tunable optical properties [20, 21]. Despite these advances, achieving near‐white lignin with high brightness remains a significant challenge.

One key limitation lies in the reactivity and selectivity of the protective agents used during lignin isolation. While alcohols such as diols are commonly employed to suppress condensation, it is hypothesized that stronger nucleophiles—such as thiols—could offer more effective protection against structural darkening. In this study, we investigate a novel fractionation method using thiolactic acid and choline chloride for lignin extraction from softwood. The thiol group in thiolactic acid is proposed to facilitate lignin fragmentation via thioacidolysis. We characterize the resulting lignin in terms of colorimetric, chemical, and morphological properties and propose a mechanistic explanation for the preservation of its light color. Finally, we demonstrate the potential of this white lignin as a light‐scattering pigment for passive radiative cooling applications.

## 2 Results

### 2.1 Fractionation of Softwood by Thiolactic Acid and Choline Chloride

**FIGURE 1:**
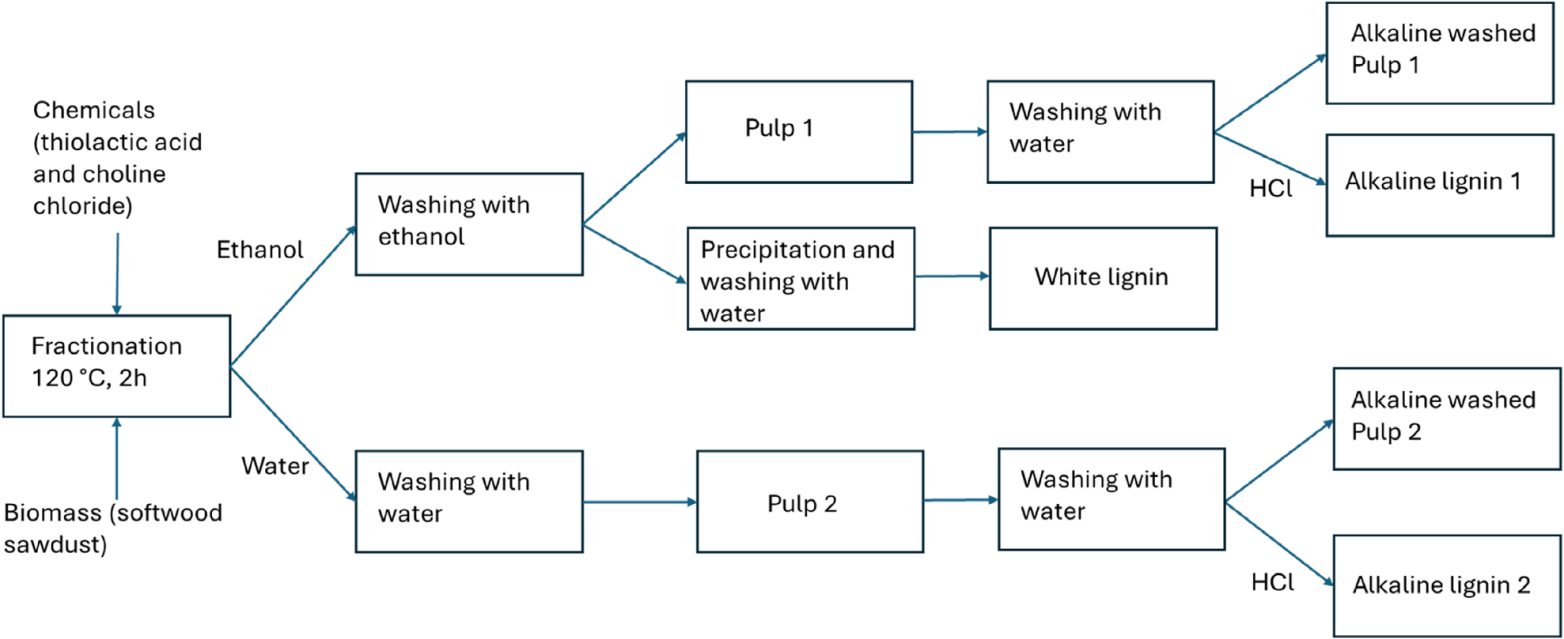
The process chart of the fractionation process.

In this study, lignocellulose biomass was fractioned using a solvent based on thiolactic acid and choline chloride to isolate white lignin. The main route for lignin isolation consists of a fractionation step followed by ethanol washing. For comparison, water was also performed. In addition to the white lignin, alkaline lignin samples were also isolated from washed pulps, followed by acid‐based precipitation. The process chart of the fractionation process is presented in Figure 1.

**FIGURE 2:**
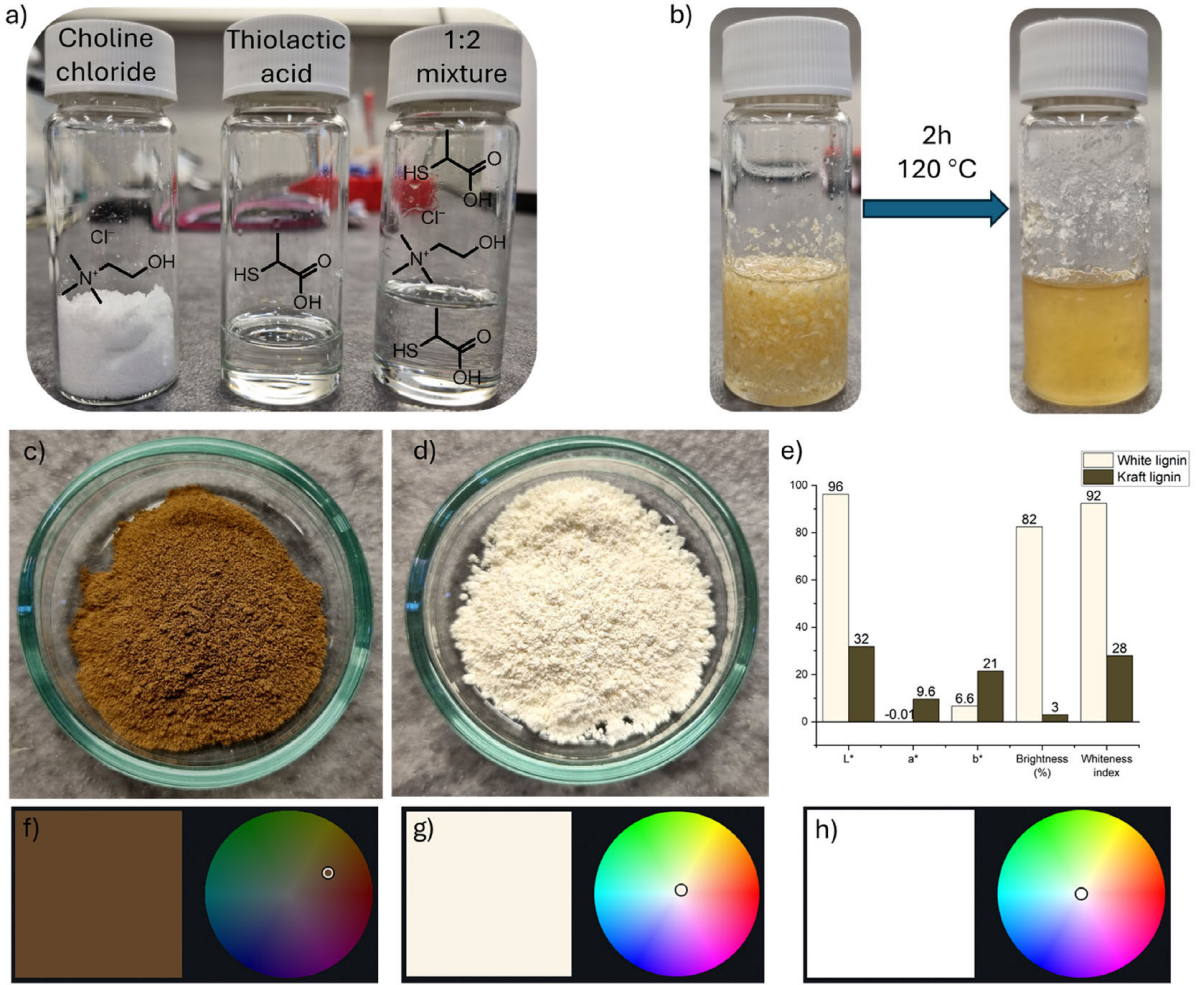
(a) Photograph of choline chloride, thiolactic acid, and their 1:2 molar ratio mixture at room temperature; (b) suspension before and after the fractionation; visual appearance of (c) kraft lignin and (d) white lignin; (e) color parameters of kraft and white lignin; visual illustration in a 2D color map of the color of (f) kraft lignin, (g) white lignin, and (h) absolute white.

The fractionation solvent was prepared by mixing choline chloride and thiolactic acid in a 1:2 molar ratio at room temperature for ≈10 min, yielding a colorless liquid (Figure 2a). This mixture resembles a well‐known DES based on choline chloride and lactic acid [22, 23]. Sawdust dispersed readily in the solvent and began to fibrillate midway through the reaction at 120°C. After 2 h, the mixture formed a slightly gel‐like, light‐yellow dispersion (Figure 2b).

Fiber liberation (i.e., fibrillation or pulping) from the original, large sawdust particles (Figure S1 and S2a) was evident in scanning electron microscopy (SEM) images of the washed solid fraction, where individual fibers were clearly visible (Figure S2b). Roundish lignin particles were observed on fiber surfaces of Pulp 1, including within pits (Figure S2c), suggesting that some dissolved lignin precipitated during ethanol washing. When water was used instead of ethanol, a significantly greater number of lignin particles were observed (Figure S2e) on the surface of Pulp 2, indicating that the choice of washing solvent influences lignin precipitation. In both cases, the precipitated lignin appeared loosely bound to the fibers and was almost completely removed by subsequent alkaline washing at room temperature (Figure S2f,g). These observations were supported by chemical analysis: lignin content decreased from ≈30 wt% in the original wood to minimal of 3.4% in alkaline‐washed Pulp 1 (Table S1).

The release of individual fibers is attributed to the dissolution of the middle lamella, which, along with outer cell wall layers, binds adjacent fibers [24]. Removal of the middle lamella facilitates fiber separation. The high fiber yield (−60 wt%) after delignification suggests minimal cellulose loss, in contrast to the kraft process, where pulp yields are typically −50 wt%. Moreover, all fiber fractions exhibited high molar mass (Mw > 300,000 g/mol) (Table S2).

A striking feature of the fiber fraction is its white appearance (Figure S2h), in sharp contrast to the brown color of unbleached kraft pulp (Figure S2i). The ISO brightness of the solid fraction was 79, nearly three times higher than that of the reference unbleached pulp (Figure S2k). The calculated whiteness index was 91. For comparison, bleached softwood kraft pulp (Figure S2j) showed only slightly higher ISO brightness (88%) and whiteness index (95) (Figure S2k). Although the exact bleaching sequence of the kraft pulp is unknown, achieving such brightness typically requires multiple bleaching stages and chlorine‐based chemicals. Thus, the thiolactic acid‐treated solid fraction demonstrates strong potential as a bleaching‐free pulp for high‐brightness applications.

While white‐colored fiber fractions are already produced industrially—albeit using hazardous bleaching agents [25]—white lignin with high lightness (L^*^ > 80) and a whiteness index near 90 has previously only been achieved through chemical functionalization of technical lignin with large quantities of hazardous isocyanates (Table 1). Remarkably, in contrast to kraft lignin (Figure 2c), the lignin precipitated from the ethanolic washing liquor exhibited a white appearance (Figure 2d), with an L^*^ value of 96 and a whiteness index of 92 (Figure 2e). Its ISO brightness was 82, compared to just 3 for kraft lignin. These results highlight the substantial differences in color properties between white lignin extracted by the thiolactic acid‐choline chloride system and kraft lignin. Notably, the color parameters of the white lignin closely matched those of the fiber fraction (Figure S2k), despite a carbohydrate content of 5 wt% in the lignin fraction (Table S4). Interestingly, previously related mercaptocarboxylic acid, thioglycolic acid has been used in lignin assay [30], resulting in the formation of darker (reddish‐brown) colored lignin [31]. However, the use of thioglycolic acid with glycolic acid allows the isolation of lignin with high whiteness [26]. This difference in lignin color may be attributed to the use of alkaline conditions during the lignin assay, where lignin is dissolved after chemical treatment and subsequently precipitated upon neutralization, leading to a more reddish coloration, also observed in this study (Table 1, second entry). The origin and impact of the precipitation method on lignin color are discussed in detail later in the text.

**TABLE 1:** Comparison of lignin yield and color parameters of different method presented in this and previous studies.

Biomass	Method/chemistry	Lignin yield	Color parameters					Reference
			L^*^	a^*^	b^*^	ISO Brightness	WI	
Softwood (Spruce)	Thiolactic acid‐choline chloride (thioacidolysis)	−70a)	96.2	−0.01	6.6	82.4	92.4	This work
Softwood (Spruce)	Thioacidolysis/Alkaline washing	−100a)	71.8	8.0	31.3	24.0	57.1	This work
Softwood (Pine)	AlCl_3_−1,4‐butane diol glycerin‐choline chloride	32	77.9	3	19.3	—	70.5	[12]
Hardwood (Poplar)	Thioglycolic acid/glycolic acid	15.5b)	84.5	—	—	—	—	[26]
Nonwood (Sugarcane)	Alkaline/Hydrogen peroxide	—	73	2.5	24.5	—	63.5	[27]
Hardwood (Poplar)	1,4‐butanediol/dioxane/HCl	52	71.5	−20	−10	22.0	63.8	[28]
Nonwood (Camellia oleifera)	Pyruvic acid	—	80.3	4.9	20.9	—	70.9	[29]
Softwood (Scotch pine)	1,4‐butanediol/citric acid (at 110C)	16	74	5.8	21.7	—	65.6	[13]
Softwood (Scotch pine)	1,4‐butanediol/citric acid (At 180C)	70	60.4	7.9	25.6	—	52.2	[13]
Nonwood (Bamboo)	Choline chloride/glycerol/AlCl_3_	64	−75	−2.5	−20	29.8	67.9	[14]
Softwood (Japanese cedar and white birch)	Enzymatic saccharification and comminution lignin (SESC)	—	66.8	8.4	19.0	—	60.9	[15]
SESC	2,6‐Diisopropylphenyl isocyanate	—	89.7	0.02	0.96	—	89.8	[15]

a) Chemical modification of lignin during the isolation is taken into account;

b) From original mass of wood.

Considering that an ideal white material would have color parameters of L^*^ = 100, a^*^ = 0, and b^*^ = 0, the white lignin produced in this study can be described as off‐white. However, as visualized in the 2D color space maps (Figure 2g,h), its color is very close to absolute white, located near the center of the color space. According to the AMS Standard 595A, the color of white lignin corresponds to White 506. Table 1 compares the colorimetric values of white lignin with those previously reported. To the best of our knowledge, lignin isolated via in current study exhibits the highest lightness and brightness reported to date.

In addition to its optical properties, the yield of white lignin—≈70% of the total lignin in wood—is notably higher than most previously reported values (Table 1). Since many lignin isolation methods do not account for chemical modifications when reporting yields, the nearly quantitative recovery achieved here, especially when combined with alkaline washing, is particularly significant. Furthermore, the thiolactic acid‐based fractionation method was also effective for hardwoods. Although the resulting hardwood lignin was slightly less white than its softwood counterpart (Table S3, Supporting Information), it still exhibited higher L^*^, whiteness index, and ISO brightness than previously reported hardwood lignin (Table 1).

Thiolactic acid can be viewed as a thiol analog of lactic acid, which, in combination with choline chloride, has demonstrated good delignification performance for lignocellulosic biomass [32, 33]. In this study, replacing thiolactic acid with lactic acid resulted in a dark‐colored lignin with a significantly lower yield (23%, Table S3). Glyoxylic acid, a related carboxylic acid with an aldehyde group, previously studied for lignin stabilization during fractionation [34, 35], produced lignin with a slightly higher yield (33%) than lactic acid, but both yield and color parameters were inferior to those obtained with thiolactic acid.

### 2.2 Chemical Characteristics of White Lignin

The functional group content of white lignin is summarized in Table 2, alongside literature data for kraft lignin and softwood milled wood lignin (MWL). The ^1^H NMR and 2D HSQC NMR spectra of white lignin are shown in Figure S3 and S4, respectively. Given the minimal variation in chemical structure among softwood lignin, the present spruce‐derived white lignin can be reasonably compared with pine MWL.

**TABLE 2:** Comparison of the main chemical groups, linkages, and degree of condensation of white lignin, kraft lignin, and milled wood lignin.

	White lignin	Kraft lignin [36]	MWL [36]
OMe	5.48	4.56	5.39
Aliphatic OH	1.56	2.83	5.94
Phenolic OH	1	3.83	1.83
Carboxylic acid	1.9	1.17	0.33
*β*‐O‐4a)	28	5	42
Degree of condensation (%)	48.1	82	43

a) per 100 aromatic unit.

White lignin contained 28 *β*‐O‐4 linkages per 100 aromatic units—lower than MWL (43) but significantly higher than kraft lignin (5). This suggests that, despite its high delignification efficiency, the thiolactic acid‐based system induces relatively mild structural degradation, preserving approximately two‐thirds of the native *β*‐O‐4 linkages. Additionally, the methoxy group (OMe) content of white lignin was comparable to that of MWL.

A particularly notable difference between white lignin and MWL is the lower content of hydroxyl (OH) groups in the former, especially aliphatic OH groups (Table 2). In contrast, white lignin exhibited a higher concentration of carboxylic acid groups than MWL. This observation suggests that thiolactic acid moieties may be covalently attached to the lignin structure, as supported by the prominent peak corresponding to the methyl group of thiolactic acid in the aliphatic region of the ^1^H NMR spectrum (Figure S3).

The weight‐average molecular weight (Mw) of the white lignin fractions was ≈43,300 g/mol (Table S2), which falls between the values reported for MWL (23,500 g/mol) and cellulolytic enzyme lignin (53,850 g/mol), and is significantly higher than typical values for softwood kraft lignin (1,700–8,000 g/mol) [37]. This relatively high Mw is consistent with the preservation of *β*‐O‐4 linkages. A comparable Mw has previously been reported for lignin isolated via aqueous acidic thiourea fractionation, where thiourea was proposed to act as a nucleophilic carbocation scavenger [38]. In that system, lignin dissolution was likely facilitated by the formation of isothiouronium intermediates, which helped suppress condensation reactions.

### 2.3 Delignification Mechanism

Thiolactic acid is a bifunctional molecule containing both carboxylic acid and thiol groups. Owing to their strong nucleophilicity, thiols have been extensively employed in thioacidolysis‐based lignin analysis [39], which typically involves the reaction of 2‐mercaptoethanol with lignin in dioxane in the presence of a Lewis acid catalyst. The thiol group can participate in various reactions with lignin, including the formation of thioacetals with carbonyl functionalities. Importantly, thiols are capable of cleaving *β*‐O‐4 and other ether linkages within the lignin polymer, leading to its depolymerization [40].

**FIGURE 3:**
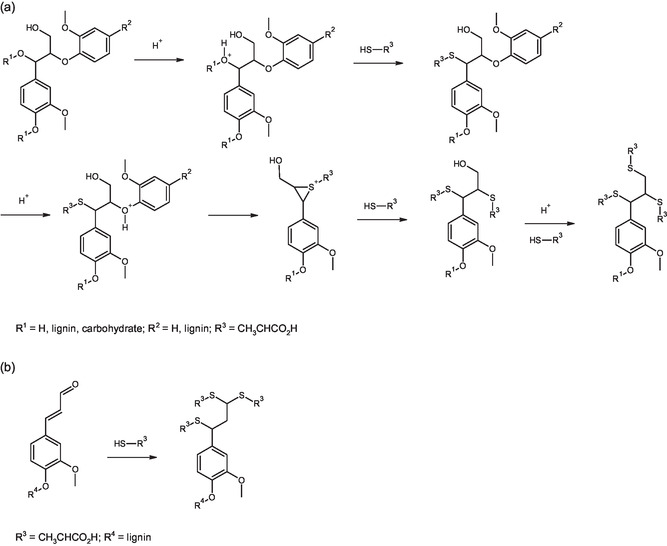
(a) Proposed mechanism for *β*‐O‐4 cleavage during thioacidolysis fractionation. Chemical composition analysis suggests that approximately one‐third of *β*‐O‐4 linkages undergo this reaction. (b) Reaction of thiolactic acid with conjugated aldehyde groups in lignin.

As discussed above, white lignin exhibited a higher content of carboxylic acid groups, a lower number of hydroxyl (OH) groups, and a reduced *β*‐O‐4 linkage content compared to MWL. Based on these observations and previously reported thioacidolysis mechanisms, we propose the reaction pathway illustrated in Figure 3a for *β*‐O‐4 cleavage during fractionation. In this mechanism, thiolactic acid (pKa = 3.74) protonates the hydroxyl group at the *α*‐position, followed by nucleophilic attack on the lignin backbone. Protonation of the ether bond facilitates fragmentation via the formation of an intermolecular episulfide ring, which is subsequently opened by another thiolactic acid molecule. Thioacidolysis is then followed by the replacement of the primary hydroxy group at the *γ* position to form the thioether moiety.

Preserving interunit linkages in lignin is generally desirable, as it is associated with a lower degree of condensation (DoC) and facilitates efficient depolymerization into monomeric products. Various lignin stabilization strategies have been developed to achieve this, with aldehydes and alcohols (e.g., diols) being among the most effective [41, 42]. Aldehydes can react with the diol structure of *β*‐O‐4 linkages to prevent dehydration, while alcohols can trap carbocations formed during acid‐catalyzed dehydration, forming more stable etherified *β*‐O‐4 linkages.

However, a trade‐off often exists between linkage preservation and lignin yield, particularly in complex biomass such as softwood. For instance, the use of citric acid and 1,4‐butanediol enabled both chemical stabilization and spatial confinement of lignin, yielding a light‐colored lignin (L^*^ = 74; Table 1) with well‐preserved *β*‐O‐4 linkages [13]—approximately half of which were converted to 1,4‐butanediol ethers—compared to MWL. Despite this, the yield of precipitated lignin was only 16% of the original Klason lignin. Increasing the temperature from 110 to 180°C significantly improved the lignin yield to − 70%, but this was accompanied by a reduction in both native and etherified *β*‐O‐4 linkages to about one‐third of the MWL value, and a darkening of the lignin color (L^*^ = 60). Therefore, despite the cleavage of interunit linkages by thiolactic acid, thioacidolysis is demonstrated to be an effective fractionation method that preserves both the yield and color of the isolated lignin.

Furthermore, as shown previously, the use of lactic acid and glyoxylic acid instead of thiolactic acid resulted in the isolation of dark‐colored lignin with inferior yield. Although lactic acid has been shown to promote the formation of etherified *β*‐O‐4 linkages [43]—similar to the thioether linkages assumed to form with thiolactic acid—the hydroxy group is notably less nucleophilic than the thiol group. Consequently, lactic acid may be less effective in preventing unwanted lignin condensation during fractionation. This condensation leads to lignin darkening and may reduce solubility, resulting in precipitation of the dissolved lignin.

In contrast, glyoxylic acid relies on aldehyde‐based protection of lignin [35], as described above. However, it lacks the nucleophilicity of thiolactic acid. Although glyoxylic acid can protect free *β*‐O‐4 linkages, the high content of benzyl ether in softwood lignin [44] may lead to carbocation formation upon cleavage of benzyl ether linkages between lignin and carbohydrates. These carbocations can promote lignin condensation.

### 2.4 Origin of the White Color of Lignin

The color of organic compounds is a complex phenomenon governed by their chemical and physical properties [45]. In organic molecules, conjugated *π*‐systems are primarily responsible for color, as electrons in *π*‐orbitals can absorb photons. Molecules with low degrees of conjugation typically absorb high‐energy photons (i.e., ultraviolet radiation), whereas increased conjugation lowers the energy gap between the highest occupied and lowest unoccupied molecular orbitals, enabling absorption in the visible range. The observed color arises from the wavelengths of visible light that are not absorbed but instead reflected, scattered, or transmitted.

According to the chemical structure of softwood MWL [46], lignin exhibits relatively low conjugation due to the prevalence of ether linkages between subunits. This structural feature aligns with the mild coloration of intact wood fibers and the presumed natural color of lignin. However, during delignification, condensation reactions can significantly increase conjugation, leading to enhanced absorption of visible light, as reflected in the high DoC of kraft lignin (82%). In contrast, the DoC of white lignin (48%) was only slightly higher than that of MWL (43%), indicating that condensation during thioacidolysis‐based fractionation is limited.

**FIGURE 4:**
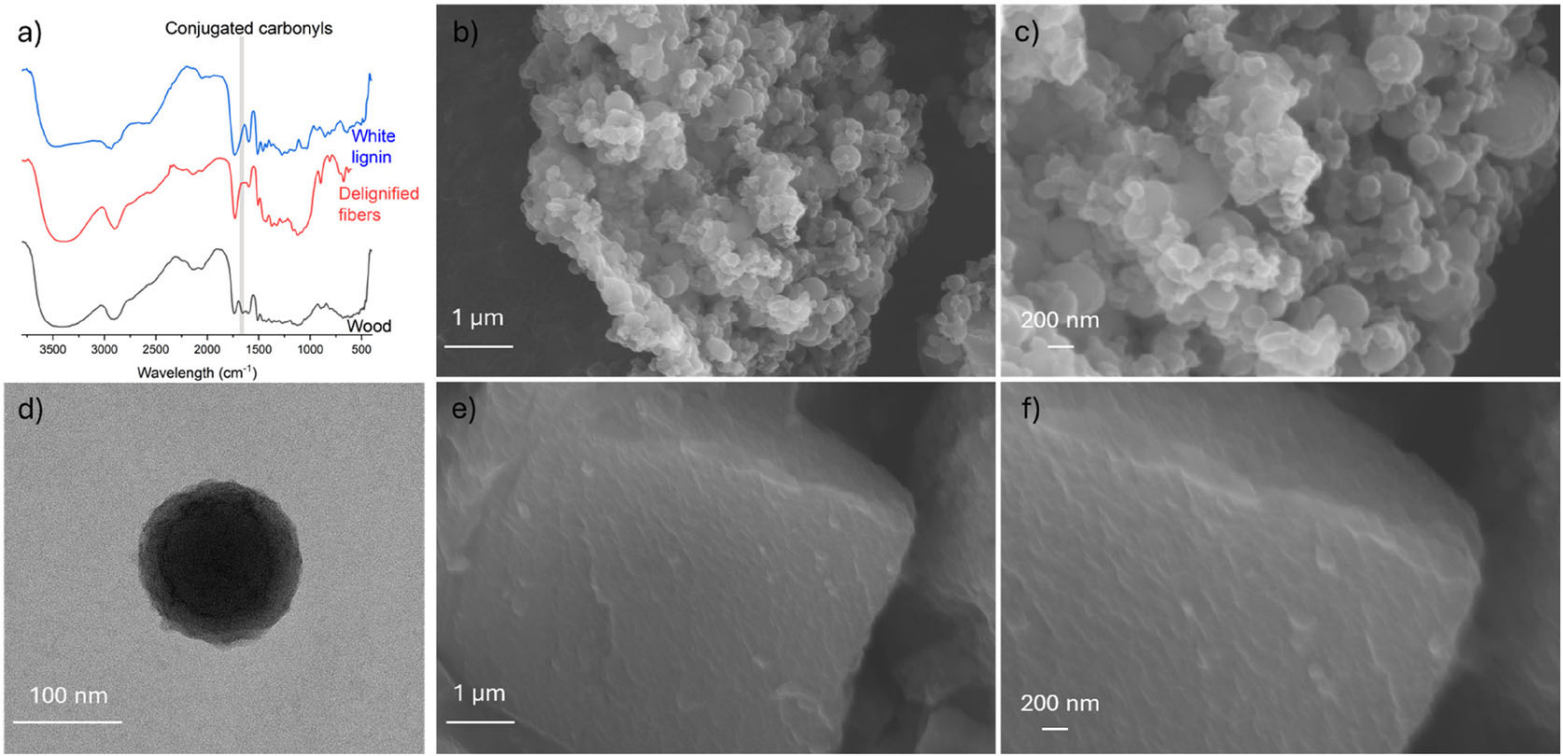
(a) Infrared spectra of original wood, delignified fibers, and white lignin showing the absence of the conjugated carbonyl peak in the thiolactic acid‐treated samples. (b) and (c) Scanning electron microscopy images of white lignin. (d) Transmission electron microscopy image of white lignin. (e) and (f) Scanning electron microscopy images of lignin precipitated from an aqueous alkaline solution.

Crucially, conjugated carbonyls—key chromophores in lignin—can be eliminated by thiolactic acid through the formation of stable thioacetals with aldehydes and ketones, and by disrupting conjugation via reactions with carbon–carbon double bonds, such as those in cinnamic aldehyde groups (Figure 3b). This is supported by the DRIFT spectra, which show the absence of the characteristic peak at −1660 cm^−1^ (associated with conjugated carbonyls) in both fiber and lignin fractions after thiolactic acid treatment (Figure 4a). Further evidence is provided by the UV spectrum of kraft lignin treated with thiolactic acid and choline chloride, which lacks the absorbance peak around 380 nm (Fig. S5). This peak is generally attributed to conjugated carbonyl and double bond structures in lignin [47].

Morphology also plays a significant role in the visual appearance of lignin. For example, lignin nanoparticles with uniform sizes can exhibit a range of colors [20]. In this study, white lignin was obtained by precipitating lignin from ethanolic washing liquor, whereas lignin precipitated from the aqueous alkaline solution used to wash the lignin‐containing treated fibers appeared yellowish, although the color of this alkaline lignin was brighter than that of kraft lignin and comparable to previously reported values (Table 1). Despite their visual differences, white and alkaline lignin exhibited nearly identical absorbance spectra in solution (Figure S6).

However, SEM imaging revealed distinct morphological differences: white lignin displayed an uneven surface composed of roundish nanoparticles of varying sizes (Figure 4b,c), also visible in TEM images (Figure 4d). In contrast, alkaline lignin exhibited a solid, relatively smooth surface (Figure 4e,f). The rough, nanostructured surface of white lignin enhances diffuse reflectance by reducing specular reflection and increasing broad‐angle backscattering, thereby contributing to its whiter appearance (Figure S7).

### 2.5 White Lignin for Passive Radiative Cooling and Solar Heating Suppression

Due to its abundance and ecofriendly properties, lignin has been proposed as a functional filler, for example, in coatings [48, 49]. The strong UV‐blocking ability of lignin renders it promising as a UV‐protecting agent; however, the strong absorption of visible light by common lignin materials causes both esthetic issues (dark color) and, more importantly, heating of lignin under solar radiation [50]. For example, a building coated with a dark coating can heat up under sunlight, which results in the need for extra cooling, thereby increasing the energy consumption and CO_2_ emissions when energy is produced from fossil‐based sources.

**FIGURE 5:**
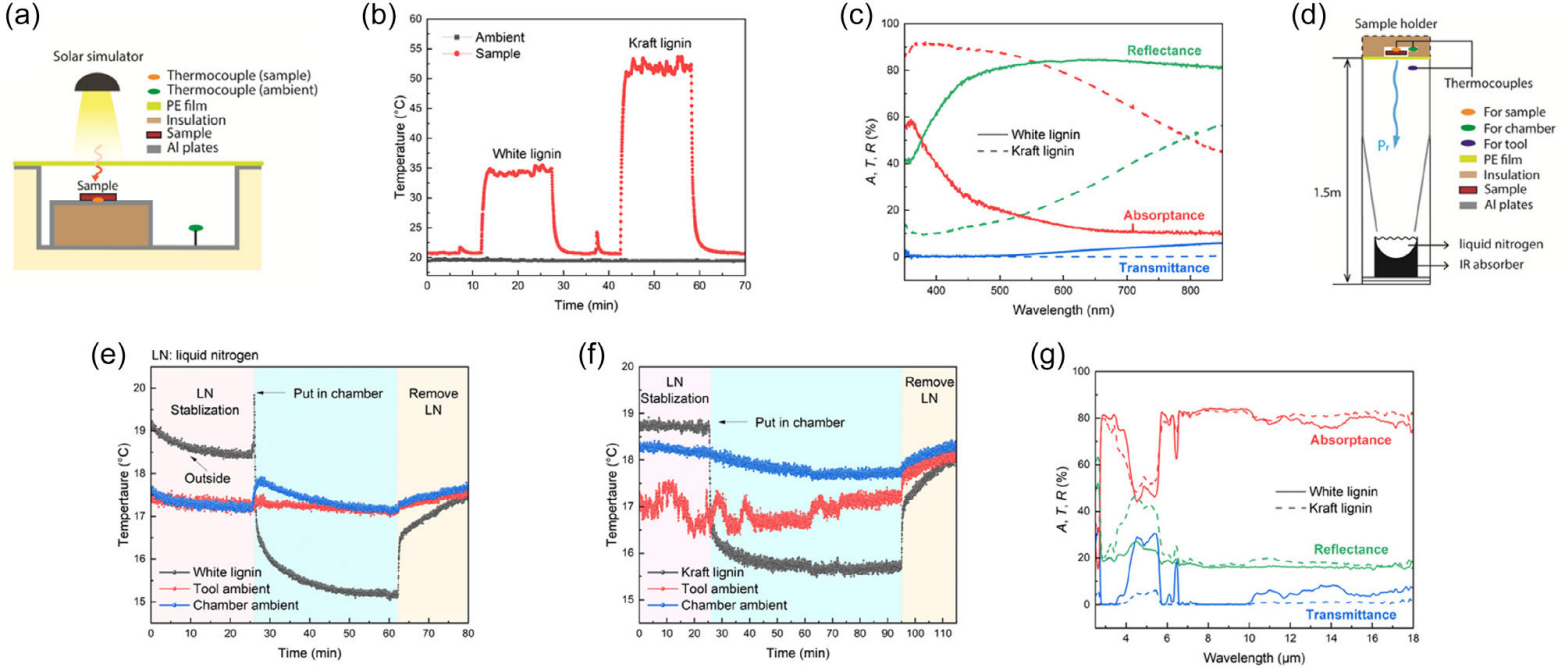
(a) Schematic of the setup used to monitor the temperature of samples exposed to light from a solar simulator. (b) Solar heating measurements for white lignin and kraft lignin samples under 1 SUN solar irradiance. (c) Total absorptance, transmittance, and reflectance of white lignin (solid lines) and kraft lignin (dashed line) in the wavelength range of 350–850 nm. d) Schematic of the sky simulator setup used to monitor the radiative cooling performance of the samples. (e) and (f) Radiative cooling measurements using a sky simulator for white lignin and kraft lignin, respectively. (g) Mid‐ and long‐range wavelength properties of white and kraft lignin films.

To demonstrate the potential of white lignin as a surface‐active compound for coatings, a model film with a white lignin content of 92 wt.% was prepared using a mixture of white lignin and cellulose nanofibers (CNFs) produced from thioacidolysis‐extracted pulp and subjected to solar irradiation (Figure 5). The results were compared with a film having 92 wt.% of kraft lignin. Thickness of white and kraft lignin films was 120 and 147 µm, respectively. Under simulated solar irradiation (Figure 5a), the white lignin sample showed an increase in temperature of only 15°C compared with ambient temperature, whereas the temperature of the kraft lignin film increased by more than 30°C (Figure 5b). This agrees with the notable visible light absorption by the kraft lignin sample (Figure 5c). In contrast, white lignin exhibited a high total reflectance of around 80% in almost the entire visible light region. White lignin showed a notable increase in absorption only at the lower end of the visible light region, which peaked in the UV region at a wavelength of around 350 nm. As the UVA region is within the range of 315–400 nm, white lignin could provide good protection against UV radiation that is not absorbed by the ozone layer. The temperature increase of white lignin upon solar irradiation is close to that reported for cellulose acetate [51].

The suppression of solar heating renders white lignin a promising material for daytime radiative cooling [52, 53]. Radiative cooling is a passive thermal management process, in which heat is dissipated by emitting long‐wave infrared radiation into cold outer space [54, 55, 56]. This mechanism benefits from the atmospheric transparency window (8–14 µm), which permits thermal radiation to escape without significant backscattering, thereby reducing the temperature of the emitting surface. When combined with low solar absorption, radiative cooling offers a sustainable alternative to conventional energy‐intensive cooling technologies.

We use a sky simulator setup (Figure 5d) to estimate the radiative cooling performance of the samples. The results indicate that both white and kraft lignin samples contribute to surface cooling (Figure 5e,f), consistent with their infrared absorptance profiles (Figure 5g). Within the atmospheric transparency window, both materials exhibit comparable absorptance, which corresponds to emissivity under Kirchhoff's law of thermal radiation [57]. These findings suggest that both lignin types can effectively emit thermal radiation. However, white lignin demonstrates greater potential as a surface coating due to its lower solar absorption, resulting in reduced temperature rise under sunlight.

Compared to other bio‐based materials such as chitin [55] and cellulose [58], lignin exhibits a slightly lower emissivity level, ≈80%. However, while both chitin and cellulose show a pronounced decrease in emissivity at the higher end of the atmospheric transparency window, lignin maintains a relatively stable emissivity across the entire 8–14 µm wavelength range. The minor drop in emissivity observed in white lignin samples between 10 and 14 µm is primarily attributed to increased transmittance, which can be mitigated by adjusting the film thickness. Furthermore, the emissivity of lignin aligns well with that of composite materials based on chitosan and melamine–phytic acid hybrid [59].

Although lignin has traditionally been considered an undesirable component in wood‐based materials for daytime passive cooling applications—often removed to enhance optical properties [60, 61]—our findings highlight its potential utility. Future studies should explore optimization of surface composition, control of physical parameters such as thickness [51], and surface modifications—such as hydrophilization—to enhance durability under environmental exposure.

### 2.6 Safety and Environmental Issues

The safety and environmental impact of solvents and reagents is a critical factor in evaluating novel chemical processes. While a comprehensive assessment—such as a life cycle analysis—is beyond the scope of this study, we provide a preliminary evaluation of the potential hazards associated with the thioacidolysis‐based method for white lignin production.

Thiolactic acid, the key reagent in this process, presents moderate toxicity. Its oral LD_50_ in rats is 730 mg/kg, making it approximately five times more toxic than, for example, lactic acid. However, both compounds exhibit similarly low dermal toxicity (>2000 mg/kg). In comparison, sodium sulfide—a common industrial pulping chemical—has a lower oral LD_50_ (208 mg/kg) and is more dermally hazardous. Importantly, thiolactic acid is readily biodegradable [62], posing minimal risk of bioaccumulation in the event of environmental release. Like most thiols, it has a pungent odor, but its relatively high vapor pressure reduces its detectability. Nevertheless, proper ventilation (e.g., use of a fume hood) is strongly recommended when handling thiolactic acid or similar compounds. In addition, proper washing is requested to eliminate the odor of thiolactic acid in white lignin. However, no characteristics smoky odor of kraft lignin, originating from volatile phenolic compounds [63], was observed in white lignin.

Unlike lactic acid, which is produced via sugar fermentation, thiolactic acid is not naturally occurring. However, it can be synthesized from bio‐based precursors such as propionic acid [64] or alanine [65] via the 2‐chloropropionic acid pathway [66] (Figure S8). Additionally, other mercaptocarboxylic acids—such as thioglycolic acid, which is synthesized from acetic acid via chloroacetic acid [67]—are promising candidates for thioacidolysis‐based fractionation to by utilized instead of thiolactic acid.

The second component of the delignification system, choline chloride, is widely regarded as a green chemical. It is a naturally occurring, biodegradable compound with low toxicity. Although industrial production currently relies on petrochemical feedstocks, a bio‐based synthesis route has been proposed [42], using ethanol derived from fermentation.

## 3 Conclusion

In summary, white lignin was isolated for the first time using a thiolactic acid‐based fractionation solvent. The mild conditions of the thioacidolysis process enabled the preservation of approximately two‐thirds of the original interunit linkages, while achieving a high lignin recovery of −70% from sawdust in a single step. The remaining lignin could be efficiently removed from the fiber fraction via mild alkaline extraction at room temperature. The resulting white lignin exhibits high reflectance in the visible spectrum and strong infrared emissivity within the atmospheric window. Combined with its UV‐absorbing properties, these features make white lignin a promising candidate for use in UV‐shielding coatings and paints with radiative cooling capabilities, as well as in sunscreens and composite materials. In addition to lignin, the process yields a cellulosic fiber fraction with brightness comparable to that of bleached kraft pulp, eliminating the need for hazardous and costly bleaching chemicals in the production of fiber‐based materials such as paper and packaging.

## 4 Methods

### 4.1 Materials

Spruce sawdust with a moisture content of 50 wt.% was kindly provided by a local sawmill (Keitele Forests Oy) and used as a raw material. Sawdust was stored in a freezer at −18°C and thawed prior to use. Thiolactic acid (≥97.0%) was obtained from TCI (Germany), lactic acid (80%) and glyoxylic acid monohydrate (98%) from Sigma–Aldrich (Germany), choline chloride (99%) from Algry Quimica, S.L. (Spain), and ethanol (96%) from VWR (USA). Deionized water was used in all the experiments. Reference kraft lignin (UPM BioPiva 100) and unbleached kraft pulp from sawdust were obtained from UMP. Bleached softwood kraft pulp was obtained from Metsä Fiber.

### 4.2 Fractionation of Sawdust

The obtained sawdust, which consisted of heterogeneously sized particles of several micrometers in size and was in an undried state close to its natural humidity (around 50 wt.%), was used without performing any size reduction treatment (e.g., grinding) or drying in order to minimize energy consumption and prevent any irreversible morphological alteration of its micro‐ and nanostructures [68].

To prepare the fractionation solution, 39.68 g of choline chloride and 49.45 mL of thiolactic acid with a molar ratio of 1:2 were measured in a Scott bottle. The mixture was stirred at room temperature until a colorless liquid was obtained, followed by the addition of 20 g of undried sawdust (10 g of dry matter). The bottle was then closed tightly with a screw cap, placed into an oil bath at 120°C, and allowed to react under mixing with a magnetic stirrer. After 2 h, the bottle was removed from the oil bath and 150 mL of ethanol was added. The mixture was then thoroughly mixed and filtered. The solid fiber fraction was then washed with 50 mL of ethanol, collected, and dispersed in 200 mL of ethanol. The dispersion was allowed to stand for 15 min with occasional manual stirring using a plastic rod. After standing, the dispersion was filtered and washed with 100 mL of ethanol. Then, the washing liquor was collected and the solid was washed with 2 L of water. The thioacidolysis‐extracted pulp was collected and stored at 4°C in an undried state.

Lignin was precipitated from the washing liquor by adding three times the amount of water relative to the volume of the ethanolic washing liquor. Precipitation was allowed to settle for around 24 h. During this time, most of the lignin accumulated on the walls of the vessel. A small amount of lignin remaining in the liquid was filtered through a polyvinylidene fluoride (PVDF) membrane (pore size 0.65 μm). Lignin was then dissolved in the minimal amount of ethanol, and three times the amount of water was added to precipitate lignin. The precipitated lignin was then filtered through a PVDF membrane (pore size 0.65 μm) and dried in a desiccator under vacuum. Water washing of the thioacidolysis‐extracted pulp was performed in a similar manner as described above except that water instead of ethanol was used for quenching the reaction and washing.

Alkaline extraction was performed on both ethanol‐ and water‐washed pulps by adding 3 g (as dry matter) of pulp into 500 mL of a 0.25 M NaOH solution. After mixing for 3 h, the fibers were filtered and washed twice with 50 mL of 0.25 M NaOH, followed by washing with 500 mL of water. The dissolved lignin was precipitated using an equimolar amount of HCl relative to NaOH. Lignin was filtered, washed with water, and dried in a desiccator under vacuum.

For comparison purposes, the fractionation was also performed in a similar manner but using choline chloride together with either glyoxylic acid or lactic acid at a molar ratio of 1:2. In addition, kraft lignin was treated with a choline chloride–thiolactic acid solution in a similar manner to that described for the sawdust.

### 4.3 Lignin‐Based Passive Radiative Cooling Film

First, the alkaline‐washed thioacidolysis‐extracted pulp was converted into CNFs via dilution into a 0.5 wt.% suspension in water and passing once through a microfluidizer (Microfluidics M‐110EH‐30, USA) equipped with 400‐μm premixing and 200‐μm interaction chambers at a pressure of 1000 bar. White lignin was then mixed with the CNF suspension to produce a mixture containing 92 wt.% of lignin. The suspension was then sonicated for 60 min using a Hielscher UP400S ultrasonic processor with a 0.5‐s pulse and a 100% amplitude in a glass beaker placed in an ice‐water bath. The suspension was then filtered through a PVDF membrane (pore size 0.65 μm) and dried between membranes and filter papers for 9 min at 93°C under a vacuum of about 70 mbar. For reference, a film using kraft lignin instead of white lignin was produced in a similar manner.

### 4.4 Characterization

The morphology of the fibers and lignin was determined via SEM (Zeiss Ultra, Germany) and transmission electron microscopy (TEM; JEOL JEM‐ 2200FS, Japan). The lignin, carbohydrate, and extractive contents of the original and acidic thiourea‐treated sawdust were determined using the National Renewable Energy Laboratory method. The color parameters and ISO brightness (ISO 2470–1) were measured in triplicate for each sample using a Konica Minolta CM‐2600d spectrophotometer (Japan). The L^*^ value ranges between 0 for black and 100 for white. The a^*^ value represents green–red opponent colors, with negative values pointing toward green and positive values pointing toward red. The b^*^ value represents blue–yellow opponent colors, with negative values pointing toward blue and positive values pointing toward yellow. The lignin structure was analyzed via 2D HSQC NMR spectroscopy using a Bruker AVANCE III 500 MHz spectrometer equipped with a 5‐mm Z‐gradient BBO (Broadband Observe) probe. For the sample preparation, 90 mg of lignin was fully dissolved in 0.6 mL of DMSO‐d6 and transferred into a 5‐mm NMR tube. The quantification of methoxy (OMe) content in lignin was performed via ^1^H NMR spectroscopy using 4‐nitrobenzaldehyde as the internal standard. In brief, 15 mg of lignin was mixed with 5 mg of 4‐nitrobenzaldehyde and fully dissolved into 0.6 mL of DMSO‐d6. After that, the mixture was transferred into a 5‐mm NMR tube and measured in a Bruker AVANCE III 500 MHz spectrometer.

The reflectance (*R*), transmittance (*T*), and absorptance (*A*) of the samples were determined by spectral directional hemispherical reflectance (DHR) and directional hemispherical transmittance (DHT). The total R, T, and A in the visible range (350–850 nm) were measured using a spectrometer (A 303iB, Andor Shamrock) equipped with a CCD detector and an integrating sphere (Labsphere). The DHR and DHT in the mid‐ and long‐wavelength infrared range were measured using a Fourier transform infrared spectrometer (Spectrum 3, Perkin Elmer) with MCT detector and an infrared‐integrating sphere. The absorptance in this range was calculated by *A* = 1 − *R* − *T*.

Solar heating measurements were conducted under 1 sun illumination using a solar simulator (Solsim LCS‐100) with normal incidence on the sample surface. The temperature evolution of the samples was continuously monitored during light exposure to evaluate their photothermal performance.

Radiative cooling measurements were carried out with a sky simulator setup (Figure 5d). The inner surface of a thermoflask was covered with black infrared‐absorbing aluminum foil (Rosco) to emulate the radiative sink of outer space. The flask was filled with liquid nitrogen to provide a cryogenic background that absorbed thermal radiation emitted from the sample. The entire chamber was constructed from polished, infrared‐reflective aluminum plates to confine and guide the radiative heat exchange. Two K‐type thermocouples (Pentronic AB) were positioned at different locations to record the sample and ambient temperatures inside the setup.

## Supporting Information

Additional supporting information can be found online in the Supporting Information section. **Supporting** **Fig. S1:** a) Photograph and b) optical microscope images of original sawdust. **Supporting** **Fig,** **S2:** Scanning electron microscope images of (a) original wood, (b) fibers after fractionation with thiolactic acid, (c) lignin precipitates in to pit of fibers, (d) lignin precipitated on the fibers by addition of water, and (f) and (g) fibers after alkaline washing. Digital photograph of (h) thioacidolysis pulp, (i) bleached kraft pulp, (j) unbleached kraft pulp from softwood. (g) color parameters of delignified wood, bleached kraft fibers, and unbleached kraft fibers (color of bar represents the color of pulp) demonstrating the extremely high brightness and color parameters of thioacidolysis pulp, being in line with bleached kraft pulp and notable higher compared to unbleached pulp. **Supporting** **Fig.** **S3:** Quantification of OME group by ^1^H NMR. **Supporting** **Fig.** **S4:** 2D HSQC NMR spectrum of the samples. (A) β‐O‐4 aryl ether linkages with a free ‐OH at the γ‐carbon; (A′) β‐O‐4 aryl ether linkages with acylated γ‐OH with p‐coumaric acid; (B) resinol substructures formed by β‐β, α‐O‐γ, and γ‐O‐α linkages; (G) guaiacyl units; (H) phydroxyphenyl units. **Supporting** **Fig.** **S5:** UV–Vis absorbance spectra of kraft lignin before (red) and after (black) thioacidolysis treatment indicating the removal of absorption peak at 380 nm related to the conjugated carbonyl (0.1 WT.% solutions in dimethyl sulfoxide). Overall color of kraft lignin was not notable changed due to the high degree of conjugation originating from kraft cooking. **Supporting** **Fig.** **S6:** UV–Vis spectra of white and alkaline lignin obtained by thioacidolysis pulping in dimethyl sulfoxide (0.1 wt.%) demonstrating their similar absorption properties. **Supporting** **Fig.** **S7:** Schematic of the scattering of light on the surfaces of white lignin and alkaline lignin. **Supporting** **Fig.** **S8:** Potential bio‐based synthetic routes to produce thiolactic acid. **Supporting** **Table S1:** Chemical constitution of original wood and different pulps obtained by thioacidolysis fractionation and alkaline washing demonstrating the high delignification efficiency of thioacidolysis pulping. The lignin content of Pulp 2 exceeds the lignin content of original sawdust due to the chemical modification of lignin, discussed in main text. **Supporting** **Table** **S2:** Molecular weight of original wood and thioacidolysis pulps with and without alkaline washing, and white lignin. **Supporting** **Table** **S3:** Lignin yield and color parameters of hardwood lignin isolated with thiolactic acid and choline chloride and softwood lignin isolated with either glyoxylic or lactic acid and choline chloride. **Supporting** **Table** **S4:** Extractive, lignin, and sugar content of white lignin.

## Funding

Business Finland (10894/31/2022 and 14/31/2023); Knut och Alice Wallenbergs Stiftelse; Wallenberg Wood Science Center and Linköpings Universitet (2009 00971).

## Conflicts of Interest

The authors declare no conflicts of interest.

## Author Contributions

**Juho Antti Sirviö** conceived the presented idea, designed and carried out the experiments, data analysis, and wrote the manuscript; **Mingna Liao** performed the solar heating and radiative cooling experiments and helped with writing; **Jasmiina Haverinen** performed composition analysis of thioacidolysis pulp; **Donya Arjmandi** and **Ruijie Wu** performed lignin and cellulose analysis and helped with writing; **Ari Ämmälä** contributed to the experimental design and writing of the manuscript; **Magnus P. Jonsson**, **Chunlin Xu**, and **Jarkko P. Räty** helped with writing.

## Supporting information

Supplementary Material

## Data Availability

The data that support the findings of this study are available from the corresponding author upon reasonable request.
