# Supplementary material for: Fractionation of Wood Biomass With Thiolactic Acid and Choline Chloride‐Based Solvent Into White Lignin for Sustainable Cooling Applications
Source: ChemSusChem. 2025 Nov 28;19(1):e202502104. doi: 10.1002/cssc.202502104 (PMC12767564; doi:10.1002/cssc.202502104)
Supplement: Supplementary file 1 — Supplementary Material [file CSSC-19-e202502104-s001.pdf]

Fractionation of Wood Biomass with Thiolactic Acid and Choline Chloride Based Solvent into White Lignin for Sustainable Cooling Applications

Juho Antti Sirviö,[1]* Mingna Liao[2,3], Donya Arjmandi[4], Jasmiina Haverinen[5], Ruijie Wu[4], Magnus P. Jonsson[2,3], Chunlin Xu[4], Ari Ämmälä,[1] Jarkko P. Räty[5]

[1] Fibre and Particle Engineering Research Unit, University of Oulu, P.O. Box 4300, 90014 Oulu, Finland E-mail: juho.sirvio@oulu.fi.

[2] Laboratory of Organic Electronics, Department of Science and Technology, Linköping University, Norrköping, SE-601 74, Sweden

[3] Wallenberg Wood Science Center, Linköping University, 601 74 Norrköping, Sweden

[4] Laboratory of Natural Materials Technology, Åbo Akademi University, Turku, 20500, Finland

[5] Kajaani University Consortium, Measurement Technology Unit, University of Oulu, Kehräämöntie 7, Kajaani, 87400, Finland

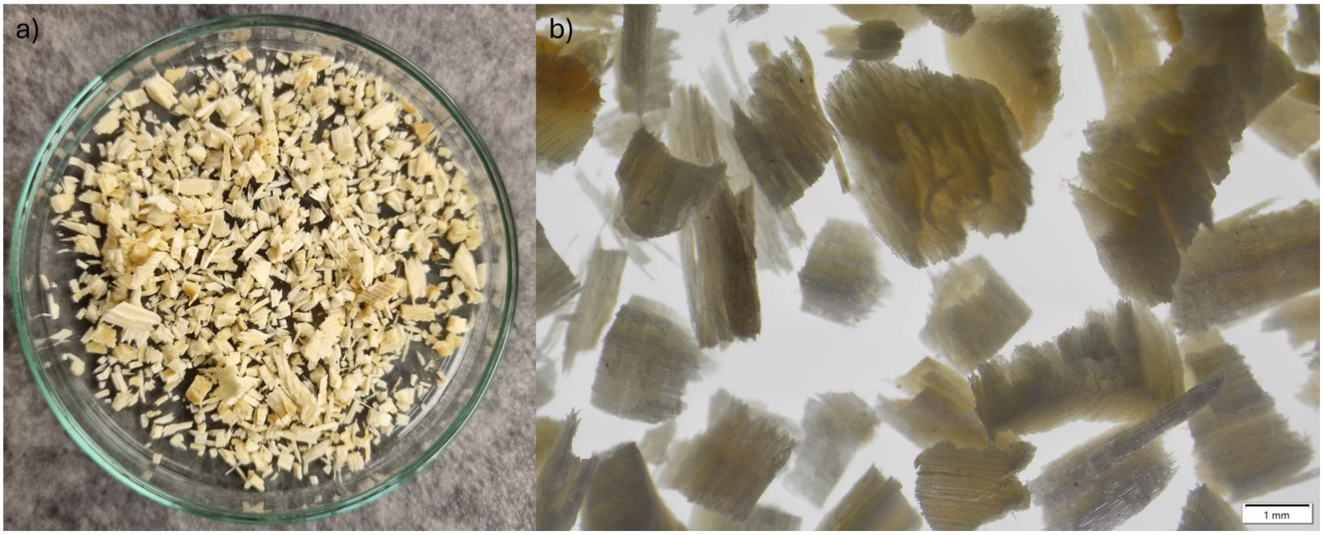

Figure 1. a) Photograph and b) optical microscope images of original sawdust

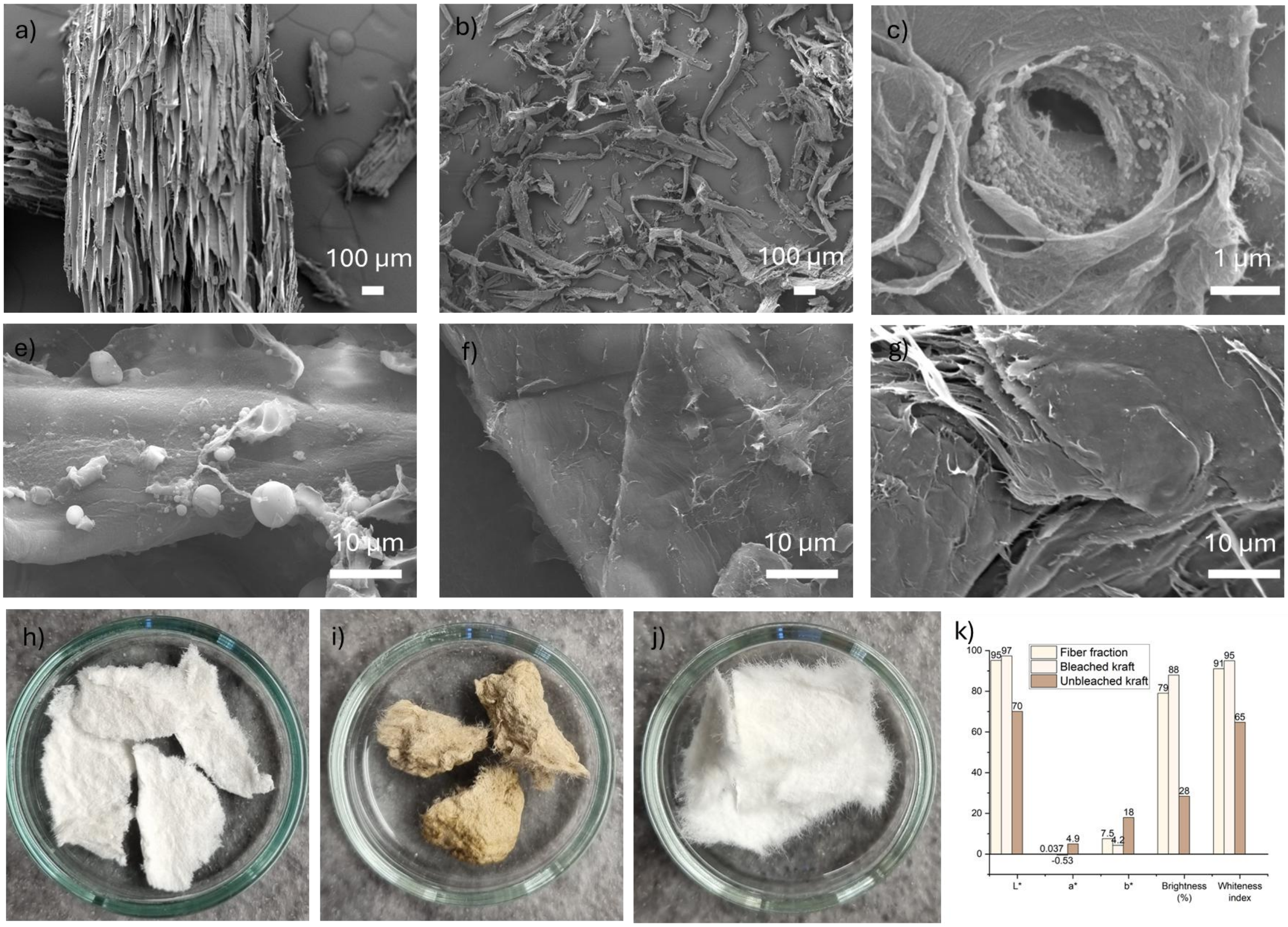

Figure S2. Scanning electron microscope images of (a) original wood, (b) fibers after fractionation with thiolactic acid, (c) lignin precipitates in to pit of fibers, (d) lignin precipitated on the fibers by addition of water, and (f) and (g) fibers after alkaline washing. Digital photograph of (h) thioacidolysis pulp, (i) bleached kraft pulp, (j) unbleached kraft pulp from softwood. (g) color parameters of delignified wood, bleached kraft fibers, and unbleached kraft fibers (color of bar represents the color of pulp) demonstrating the extremely high brightness and color parameters of thioacidolysis pulp, being in line with bleached kraft pulp and notable higher compared to unbleached pulp.

Table S1. Chemical constitution of original wood and different pulps obtained by thioacidolysis fractionation and alkaline washing demonstrating the high delignification efficiency of thioacidolysis pulping. The lignin content of Pulp 2 exceeds the lignin content of original sawdust due to the chemical modification of lignin, discussed in main text.

	Glucose (%)	Xylose (%)	Galactose (%)	Mannose (%)	Acid insoluble lignin (%)	Acid soluble lignin (%)	Total lignin content (%)
Sawdust	46.9	4.23	1.8	11.0	28.6	1.85	30.5
Pulp 1[a]	71.7	-[c]	-[c]	5.4	10.0	3.1	13.1
Alkaline washed Pulp 1	88.5	-[c]	-[c]	6	1.9	1.5	3.4
Pulp 2[b]	55.0	5.6	-[c]	3.7	33.6	5.5	39.1
Alkaline washed Pulp 2	67.4	-[c]	-[c]	5.8	5.7	1.7	7.4

[a] Pulp washed with ethanol, [c] Pulp washed water, [c] The amount below detection limits

Table S2. Molecular weight of original wood and thioacidolysis pulps with and without alkaline washing, and white lignin.

	Mw (g/mol)	Mn (g/mol)	Polydispersity
Sawdust	802	340	2.36
Pulp 1[a]	464	98.5	4.71
Alkaline washed Pulp 1	313	74.0	4.23
Pulp 2[b]	421	132	3.19
Alkaline washed Pulp 2	314	59.5	5.28
White lignin	43.3	7.8	5.55

[a] Pulp washed with ethanol, [c] Pulp washed water

Table S3. Lignin yield and color parameters of hardwood lignin isolated with thiolactic acid and choline chloride and softwood lignin isolated with either glyoxylic or lactic acid and choline chloride.

Biomass	Method/chemistry	Lignin yield	Color parameters				
			L*	a*	b*	ISO Brightness	WI
Hardwood (Birch)	Thiolactic acid-choline chloride (thioacidolysis)	22.5[a]	81.6	5.1	14.4	46.4	76.1
Softwood (Spruce)	Glyoxylic acid-choline chloride	10.5	33.7	9.3	22.5	3.3	29.4
Softwood (Spruce)	Lactic acid-choline chloride	23.0	41.2	9.7	20.9	6.1	36.9

[a]From original mass of wood

Table S4. Extractive, lignin, and sugar content of white lignin

	Chemical composition (%)										
	Extractives	Acid insoluble lignin	Acid soluble lignin	Xylene	Mannose	Glucose	4-O-methylation of glucuronic acid	Galactose	Galacturonic acid	Rhamnose	Total sugar
White lignin	4.13	86.4	2.9	0.83	0.85	0.85	0.03	0.85	0.85	0.85	5.1

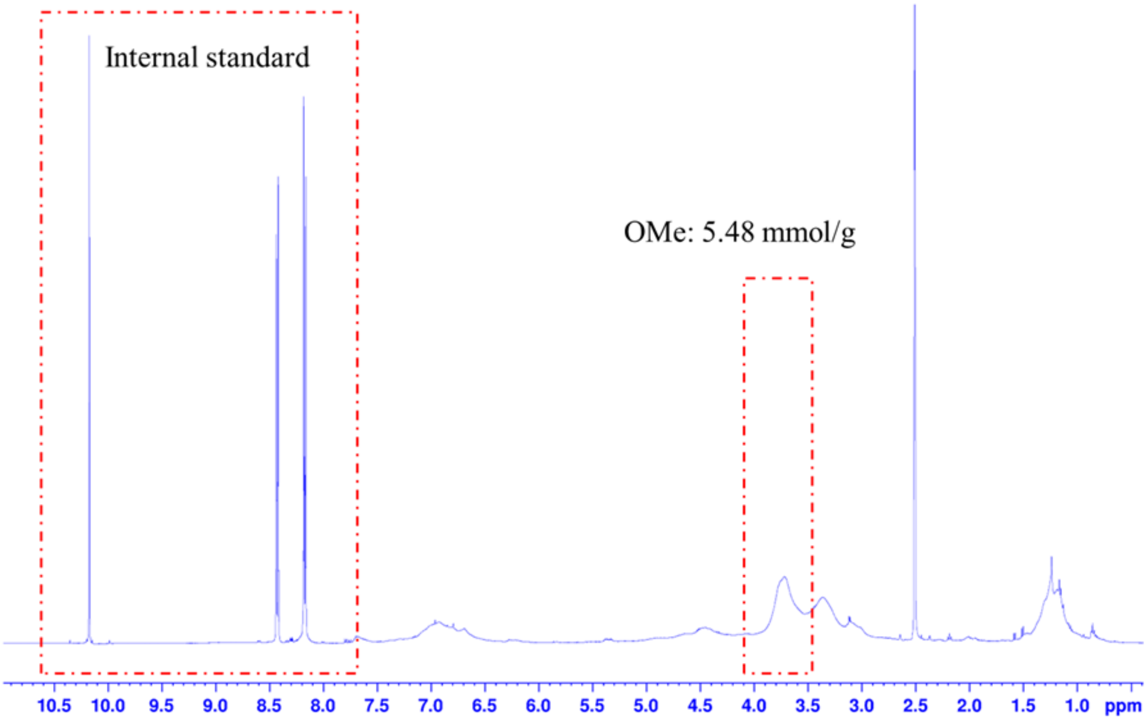

Figure S3. Quantification of OME group by ^{1}H NMR.

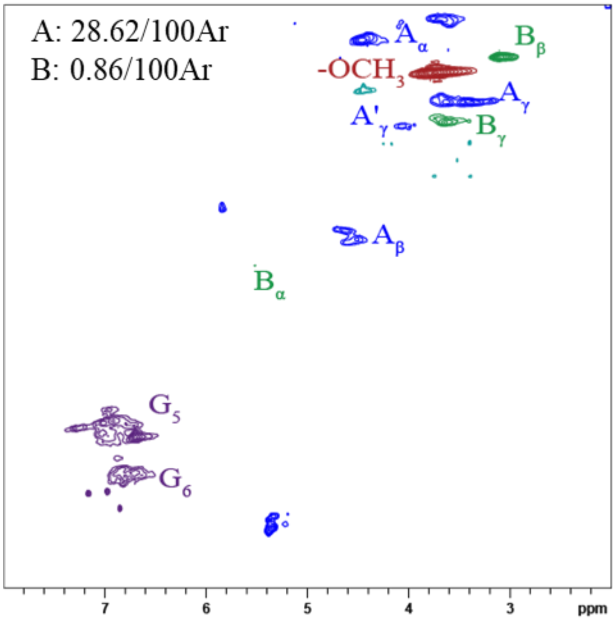

Figure S4. 2D HSQC NMR spectrum of the samples. (A) β-O-4 aryl ether linkages with a free -OH at the γ-carbon; (A′) β-O-4 aryl ether linkages with acylated γ-OH with p-coumaric acid; (B) resinol substructures formed by β-β, α-O-γ, and γ-O-α linkages; (G) guaiacyl units; (H) phydroxyphenyl units.

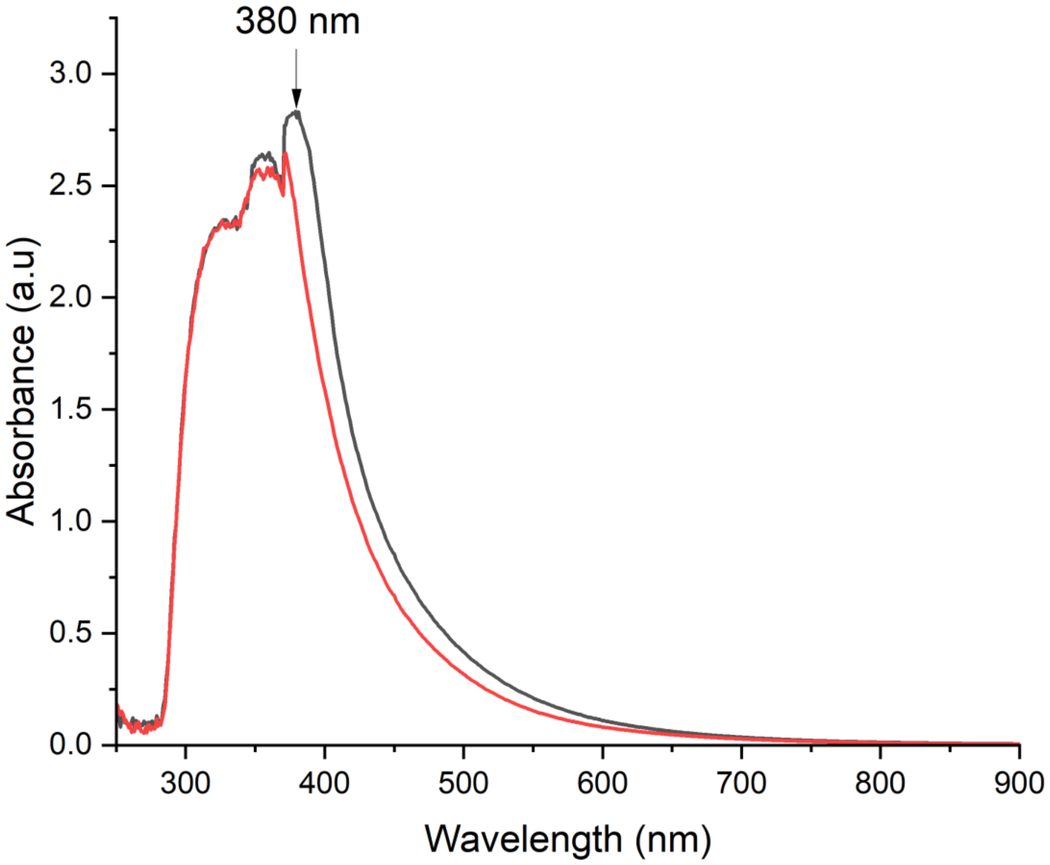

Figure S5. UV-Vis absorbance spectra of kraft lignin before (red) and after (black) thioacidolysis treatment indicating the removal of absorption peak at 380 nm related to the conjugated carbonyl (0.1 WT.% solutions in dimethyl sulfoxide). Overall color of kraft lignin was not notable changed due to the high degree of conjugation originating from kraft cooking.

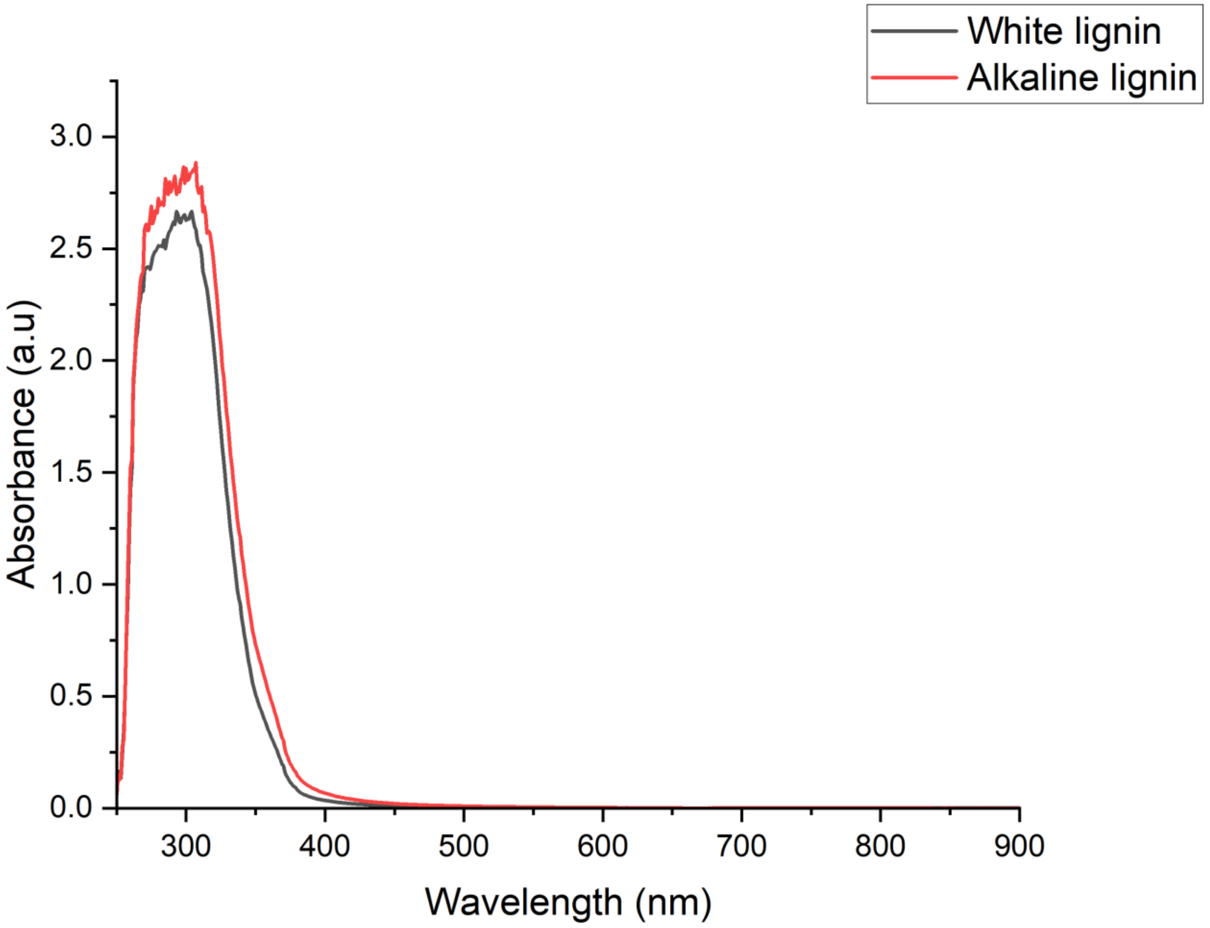

Figure S6. UV-Vis spectra of white and alkaline lignin obtained by thioacidolysis pulping in dimethyl sulfoxide (0.1 wt.%) demonstrating their similar absorption properties.

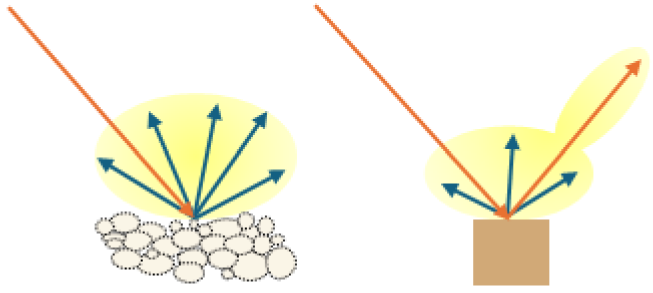

Figure S7. Schematic of the scattering of light on the surfaces of white lignin and alkaline lignin.

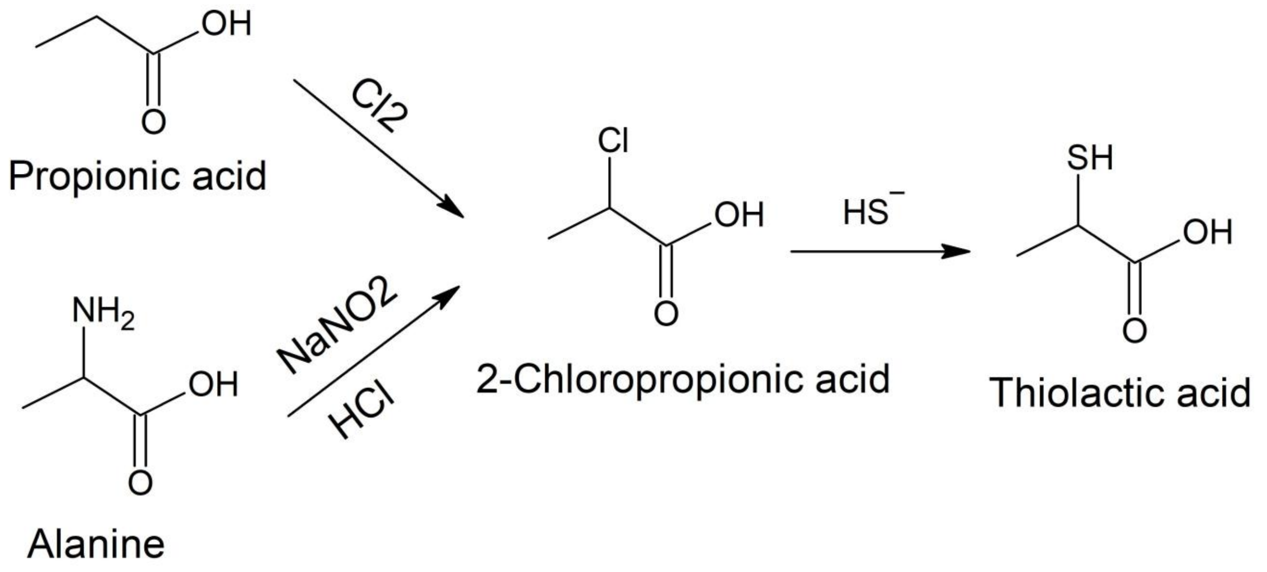

Figure S8. Potential biobased synthetic routes to produce thiolactic acid.
